# Utility of Tissue Transglutaminase Immunohistochemistry in Pediatric Duodenal Biopsies: Patterns of Expression and Role in Celiac Disease—A Clinicopathologic Review

**DOI:** 10.1155/2013/602985

**Published:** 2013-09-08

**Authors:** Saeeda Almarzooqi, Ronald H. Houston, Vinay Prasad

**Affiliations:** ^1^Nationwide Children's Hospital, 700 Children's Drive, Columbus, OH 43205, USA; ^2^Anatomic Pathology ChildLab, Nationwide Children's Hospital, 700 Children's Drive, Columbus, OH 43205, USA; ^3^Nationwide Children's Hospital, Clinical Ohio State University, 700 Children's Drive, Columbus, OH 43205, USA

## Abstract

Tissue transglutaminase (tTG) is a ubiquitous multifunctional protein. It has roles in various cellular processes. tTG is a major target of autoantibodies in celiac disease, and its expression by immunohistochemistry in pediatric celiac disease has not been fully examined. We studied tTG expression in 78 pediatric duodenal biopsies by utilizing an antibody to transglutaminase 2. Serum tTG was positive in all celiac cases evaluated. Serum antiserum endomysial antibody (EMA) and tTG were negative in all control subjects and in inflammatory bowel disease and eosinophilic gastroenteritis. There was a statistically significant difference between cases of celiac disease and normal controls in terms of tTG immunohistochemical staining in duodenal biopsies surface epithelium (*P* value = 0.0012). There was no significant statistical difference in terms of staining of the villous surface or crypt between the cases of celiac disease and cases with IBD (*P* value = 0.5970 and 0.5227, resp.). There was no detected correlation between serum tTG values and immunohistochemical positivity on duodenal biopsy in cases of celiac disease (*P* value = 1). There was no relationship between Marsh classification and positivity of villous surface for tTG (*P* value = 0.4955). We conclude that tTG has limited utility in diagnosis of celiac disease in pediatric duodenal biopsies.

## 1. Introduction

 The diagnosis of celiac disease is based on demonstrating characteristic villous abnormality on duodenal biopsy in a patient with positive celiac serology. Among the serological tests used are antiserum endomysial antibody (EMA) and antitissue transglutaminase antibodies (tTG). 

EMA antibodies have a sensitivity of 92.1% and a specificity of 99.8% [1]. EMA antibodies are of the IgA subtype, and thus, false negative cases may be encountered in patients with IgA deficiency [2]. Serum tTG has a sensitivity of 94.8% and specificity of 99.2% [1].

Tissue transglutaminase is an intracellular enzyme present in many tissues. It has roles in various cellular processes including cellular differentiation, matrix production, and tissue repair [3, 4]. Serum tTG has been found to be increased in patients with celiac disease [3]. It has been confirmed that this enzyme is a target in the autoimmune process of celiac disease that is targeted by EMA [1, 5]. tTG deamidates glutamine residues of the glutamine-rich gliadins exposing them and facilitating their presentation by antigen presenting cells to T cells [6].

The validity of utility of tTG immunohistochemistry in duodenal biopsy of celiac disease has conflicting results in the literature, some authors finding it to be a useful test in differentiating celiac disease cases from normal control, while others have failed to demonstrate such usefulness [2, 4, 6]. In addition, its expression by immunohistochemistry in pediatric celiac disease has not been fully examined. The aim of our study was to investigate the pattern of expression of tTG in pediatric patients with celiac disease and compare it with normal duodenal biopsy and patients with other duodenal pathology, for example, inflammatory bowel disease (IBD).

## 2. Materials and Methods

We obtained approval from the Institutional Review Board (IRB) of Nationwide Children's Hospital to undertake this study. 78 cases of duodenal biopsy from the files of the Department of Anatomic Pathology were retrieved; 36 celiac disease, 33 pathologically normal controls, 7 inflammatory bowel disease, and 2 eosinophilic gastroenteritis.

 Three micron-thick sections of formalin-fixed, paraffin-embedded duodenal biopsies were cut and mounted on charged slides. An antibody to tissue transglutaminase, clone CUB 7402 (Abcam Inc., Cambridge MA), was used at a dilution factor of 1 : 1280, after antigen retrieval in a citrate buffer, pH 6 (ER1; Leica Microsystems, Buffalo Grove, IL). A negative control was obtained by substituting the primary antibody with Universal Negative Control Serum (cat no. NC49BL, Biocare Medical, Concord CA). All stages of the immunohistochemistry procedure were automated on the BondMax IHC system (Leica Microsystems) using the Refine polymer detection system (Leica Microsystems), with DAB visualization and counterstained with hematoxylin.

Laboratory serological studies for Serum IgA endomysial antibodies (EMA) or tissue tansglutaminase IgA antibodies (tTG) were obtained by review of medical charts. 

Results were evaluated by using Fisher's exact test with SAS software (SAS 9.1.3 SAS 9.1.3 by SAS Institute Inc., Cary, NC, USA).

## 3. Results

A total of 78 cases were evaluated. The diagnosis of celiac disease (36 patients) had been confirmed based on clinical, serological, and histological evaluation. All the cases were biopsied because of clinical symptoms of pain. None of the cases of celiac disease that were biopsied were on a Gluten-free diet. 7 cases of IBD (5 Crohn's disease, 2 ulcerative colitis) were also retrieved. The cases of inflammatory bowel disease did not have small bowel involvement at the time the biopsies were taken. Two patients had eosinophilic gastroenteritis. In addition, 33 cases of histologically normal duodenal biopsies that underwent upper gastrointestinal (GI) endoscopy for evaluation of upper GI symptoms were included. The clinical characteristics of our patients are summarized in Table 1. Among the group with celiac disease there were 23 females and 13 males. The age range was 1–16 years (median 8 years). Comorbidity was noted in a number of the cases and included diabetes mellitus (7), Down syndrome (2), juvenile rheumatoid arthritis (1), and hypothyroidism (1). 

In patients with celiac disease, serum tTG was positive in all cases where it was evaluated (26/26). There were 10 cases in which serum tTG was not evaluated before or at the time of biopsy, and these cases were lost to follow up. Therefore, the results of serum tTG testing were available only in 26 cases. Serum EMA was elevated in 26/30 and was not elevated in 4/30, Table 2(b). In the remaining 6 cases, serum EMA was not evaluated. Serum EMA and tTG were negative in all control subjects and in cases with inflammatory bowel disease and eosinophilic gastroenteritis.

**Figure 1:**
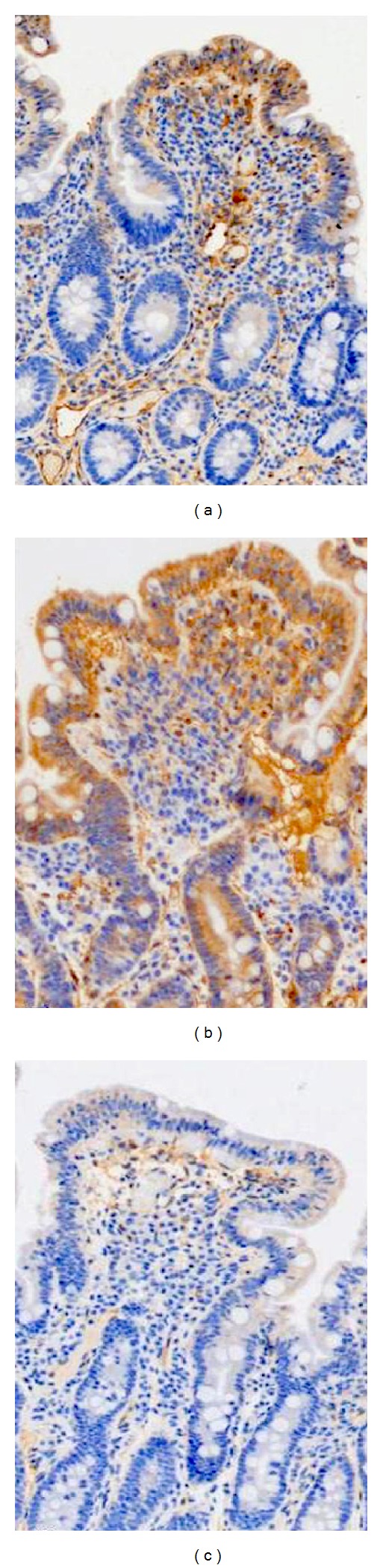
Patterns of expression of tissue transglutaminase in duodenal biopsies of celiac disease. (a) Positive surface mucosa labelling with absent staining in crypts. (b) Positive staining in both surface mucosa and in crypts. (c) Absence of staining in both surface epithelium and crypts. (immunoperoxidase 20x).

 Among the cases with celiac disease, the pattern of mucosal staining with tTG was characterized by one of three staining patterns, Table 2(a) and Figure 1. The majority of celiac cases (27/36) demonstrated both surface epithelial staining with localization to enterocyte cytoplasm, superficial lamina propria, and basement membrane. This pattern was accompanied by an absence of staining in mucosal crypts. Three of the cases showed both surface epithelial positivity as well as crypt staining. Absence of staining was noted in six of the celiac cases. 

**Figure 2:**
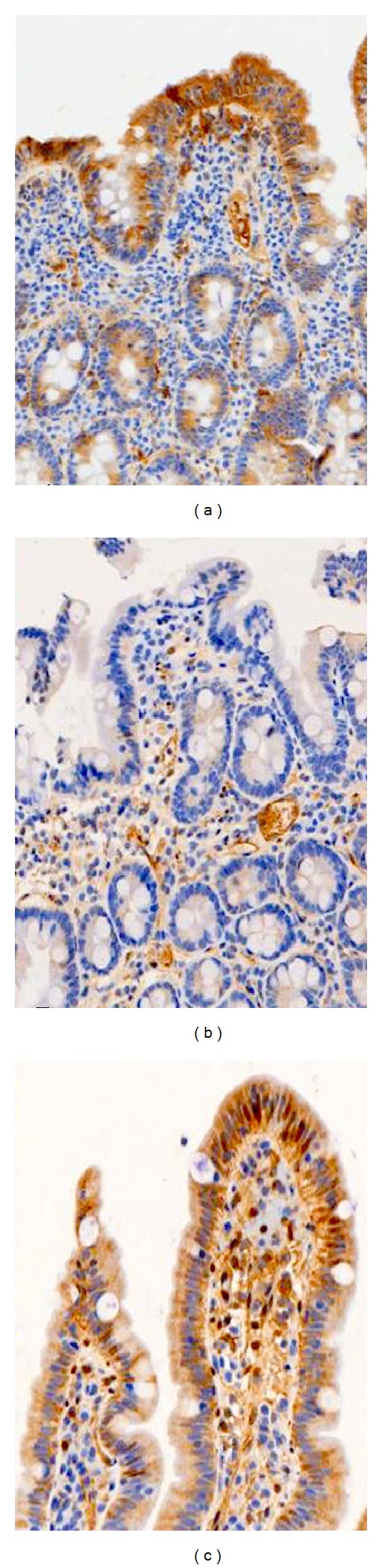
Expression of tissue transglutaminase in duodenal biopsies. (a) Celiac disease. (b) Normal control. (c) Crohn's disease. (immunoperoxidase 20x).

Figure 2 demonstrates the predominant patterns of expression of tTG among cases of celiac disease, normal control, and cases with inflammatory bowel disease. When comparing all groups for the pattern of tTG staining, there was a statistically significant association between the different groups and tTG villous surface staining (*P* value = 0.0045). There was no significant association between different groups and tTG staining in duodenal crypts (*P* value = 0.7272). There was a statistically significant difference between celiac cases and normal control in terms of tTG immunohistochemical staining in duodenal biopsies surface epithelium (*P* value = 0.0012). No difference was present between immunohistochemical staining of crypt epithelium (*P* value = 1) (Table 3).

There was no significant difference in terms of villous surface staining between celiac disease cases and cases with IBD (*P* value = 0.5970). Similarly, there was no significant difference between crypt staining with tTG between the two groups (*P* value = 0.5227) (Table 4). Combining the cases of eosinophilic gastroenteritis with cases of IBD failed to detect any significant difference between them and patients with celiac disease by evaluation staining in surface villi and crypt (*P* value = 0.3536 and 1, resp.).

There was no detected correlation between serum tTG values and immunohistochemical positivity on duodenal biopsy in cases of celiac disease (*P* value = 1). Categorizing cases of celiac disease using the Marsh classification yielded one case of class 0, 5 cases of class 2, 10 cases of class 3a, 19 cases of 3b, and 1 case of class 3c. There was no relationship between Marsh type and positivity of villous surface for tTG (*P* value = 0.4955) (Table 5).

The results of this small study are significant because we did not observe a specific single pattern of staining. We observed immunohistochemical staining with tTG in different areas within the biopsies, such as crypt epithelium, lamina propria, and basement membrane. The results also showed that there is no significant difference in the pattern of tTG staining among cases of celiac disease versus chronic duodenitis due to inflammatory bowel disease. Additionally, a few cases of celiac disease had no staining with immunohistochemical stain for tTG. In this study, the Marsh grades noted and the tTG staining patterns also did not follow a pattern.

## 4. Discussion

Tissue transglutaminase is an intracellular enzyme present in many tissues. It has roles in various cellular processes including cellular differentiation, matrix production, and tissue repair [3, 4]. Serum tTG has been found to be increased in patients with celiac disease [3]. It has been confirmed that this enzyme is a target in the autoimmune process of celiac disease that is mediated by endomysial antibodies [1, 5].

The utility of tTG immunohistochemistry in the evaluation of duodenal biopsy for the diagnosis of celiac disease has been reported recently [2–4]. However, results of the different studies had conflicting results in terms of the specificity of the immunohistochemical test in differentiating celiac disease from nonceliac cases [2–4].

Serological screening for celiac disease is based on identification of serum antiendomysial IgA antibodies. The test has a sensitivity and specificity >95% [2]. Limitations encountered with the utility of this paper include lower sensitivity (<90%) in patients below the age of 2 years and the false negativity seen in patients with IgA deficiency [2, 7]. This has been overcome by the measurement of serum IgA in all patients undergoing serological evaluation for celiac disease. Serum tTG levels have a sensitivity and specificity up to 98% [2].

In our series of celiac disease, the findings are in accordance with those reported in the literature. All of our patients who have been tested for serum tTG had a positive result. Of those patients evaluated for serum EMA, 26 out of 30 patients had an elevated serum level. Thus, according to our data, serum EMA has a sensitivity of 86% and a specificity of 100%. 

The patterns of localization of tTG in celiac duodenal biopsies were characterized by a predominant surface epithelial staining with absent staining in mucosal crypts. The staining was located in the cytoplasm of enterocytes as well as in superficial lamina propria and subepithelial basement membrane. This pattern was noted in 75% of our celiac disease cases. Three of our cases (3/36) showed both surface epithelial staining as well as staining of crypts. In six cases (6/36), no staining was detected. Among these cases with a negative staining pattern, a positive serologically elevated serum tTG was seen in the four cases where it was measured. Previous studies have shown the patterns of tTG localization in celiac disease duodenal biopsy. Salky et al. have demonstrated that localization of tTG in duodenal mucosa was related to degree of mucosal atrophy. In their series, cases of celiac disease showed an increased expression of the tTG in the basement membrane and lamina propria when compared to control cases. They suggest that this pattern of localization can be explained on the basis that tTG forms complexes with gliadin in the basement membrane and lamina propria in celiac patients [8]. Similar results have been shown by other authors as well [4, 6].

Our data demonstrate that the utility of immunohistochemical tTG in evaluation of duodenal biopsy for suspected celiac disease has a significant value when comparing it to normal controls. Esposito et al. evaluated the expression of tTG in 26 patients (0.9–18 years) and showed an increased expression in duodenal biopsies of their cases. They showed increased staining in subepithelial connective tissue as well as in enterocytes in cases of celiac compared with control subjects. They suggest that the staining is either disease specific or secondary to inflammation [4]. Similar studies were also in support of the utility of this immunohistochemical stain in differentiating celiac disease from normal control [6, 8]. However, Tuncer and colleagues in a review of 12 cases of celiac disease failed to demonstrate a significant difference in comparison to normal controls [2].

 A limitation of this study is the possibility of low detectability, perhaps due to technical constraints, leading to the minimal staining within the lamina propria in some cases. We also noted a subset of patients with celiac disease who had no immunohistochemical tTG staining, a confounding factor. Another limitation of this immunohistochemical stain is the demonstration of mucosal positivity in cases of duodenitis due to other etiologies. In our series, seven cases of inflammatory bowel disease and two cases of eosinophilic gastroenteritis were evaluated. We failed to demonstrate any significant statistical difference. This observation has been noted by other investigators [4, 6, 9]. In the series by Gorgun and colleagues, cases of duodenitis other than celiac disease were evaluated. Their cases included one case of Crohn's disease, 5 cases of acid related duodenitis, one case of Campylobacter duodenitis, one case of abetalipoproteinemia, and one case of Waldenström macroglobinemia. Their data failed to show any difference when comparing these cases to cases of celiac disease [6]. Similarly, Esposito et al. demonstrated the expression of tTG in one patient of Crohn's disease. They suggested that the positivity might not be disease specific but rather a marker of an abnormal immune response [4].

 Immunohistochemical staining in cases of celiac disease does not correlate with the Marsh type. 80% of the cases were Marsh types 3a or 3b. 

 In conclusion, the immunohistochemical utility of tTG in celiac duodenal biopsies seems to be of limited value. This is due to expression in inflamed duodenal mucosa in nonceliac cases such as cases of inflammatory bowel disease. This immunohistochemical stain has to be studied in more detail, in a larger number of children. It would also be beneficial to examine the tTG staining of patients who are on gluten-free diets and compare the findings. Therefore, one should not rely on immunohistochemical expression of tTG to make a diagnosis of celiac disease.

## Figures and Tables

**Table 1:** Clinical characteristics of subjects.

Group (*n*)	Age range (median)	Gender		Other diseases (*n*)	Clinical symptoms
		F	M		
Celiac disease (36)	1–16 (8)	23	13	DM (7) Down syndrome (2) Juvenile RA (1) Hypothyroidism (1)	(i) Abdominal pain (ii) Diarrhea (iii) Failure to thrive/weight loss (iv) Constipation

IBD (7)	5–17 (15)	2	5		(i) Rectal bleeding (ii) Diarrhea (iii) Abdominal pain (iv) Weight loss

*Normal control (33)	0.6–17 (8)	16 17		Asthma (5) Juvenile RA (1)	(i) GERD (ii) Abdominal pain

Eosinophilic GE (2)	1–11 (6)	2			(i) Dysphagia (ii) Vomiting

*n*: number; IBD: inflammatory bowel disease; GE: gastroenteritis; F: female; M: male; DM: diabetes mellitus; RA: rheumatoid arthritis; GERD: gastroesophageal reflux disease; *diagnoses of control cases were 17 cases with no pathologic diagnosis, 7 chronic gastritis, 6 active esophagitis, 2 reactive gastritis, and 1 candida esophagitis.

**Table:** (a)

Pattern of expression	tTG surface positive/crypt negative	tTG surface positive/crypt positive	tTG surface/crypt negative

No. of cases (total 36)	27	3	6

**Table:** (b)

	Serum tTG elevated	Serum EMA elevated	Serum not tested
No. of cases (total cases 36)	26 of 26 tested	26 of 30 tested (4/30 not elevated)	6 not tested for EMA 10 not tested for serum tTG

**Table 3:** Immunohistochemical staining pattern in celiac disease and control cases.

	Surface tTG		Crypt tTG	
	Positive *n* (%)	Negative *n* (%)	Positive *n* (%)	Negative *n* (%)
Celiac disease	30 (83.33)	6 (16.67)	3 (8.33)	33 (91.67)
Normal control	15 (45.45)	18 (54.55)	2 (6.06)	31 (93.94)
*P* value	0.0012		1.0	

*n*: number; %: percentage.

**Table 4:** Immunostaining pattern in celiac and inflammatory bowel disease.

	Surface tTG		Crypt tTG	
	Positive *n* (%)	Negative *n* (%)	Positive *n* (%)	Negative *n* (%)
Celiac disease	30 (83.33)	6 (16.67)	3 (8.33)	33 (91.67)
IBD	5 (71.43)	2 (28.57)	1 (14.29)	6 (85.71)
*P* value	0.5970		0.5227	

*n*: number; %: percentage; IBD: inflammatory bowel disease.

**Table 5:** Cases of celiac disease subclassified by Marsh classification.

Marsh classification	No. of cases
0	1
2	5
3a	10
3b	19
3c	1
